# Identifying diseases in claims data using a machine learning approach – a case from Switzerland

**DOI:** 10.1186/s13690-025-01813-y

**Published:** 2025-12-30

**Authors:** Michael Stucki, Andreas Kohler, Stefan Boes

**Affiliations:** 1 ZHAW Zurich University of Applied Sciences, Winterthur Institute of Health Economics, Winterthur, Gertrudstrasse 8 8401 Switzerland; 2 Faculty of Health Sciences and Medicine, University of Lucerne, Lucerne, Switzerland

## Abstract

**Background:**

In many health care systems, health data often include either diagnostic or cost data but not both. This poses a challenge for epidemiological or cost-of-illness studies. In this paper, we aim to identify diseases based on the utilization of outpatient services employing statistical learning models.

**Methods:**

We combine insurance claims data of the hospitalized population of a large Swiss health insurer from 2017 with diagnostic information in the national hospital inpatient registry from 2016 and 2017 at the patient level. We use random forests and boosting algorithms to predict the presence of 32 diseases based on outpatient health care utilization alone. The features include drug spending by four-digit ATC codes, spending by service provider, and spending by subchapter of national fee-for-service catalogues. We use the models to predict the prevalence of a disease in the non-hospitalized population for which no disease labels are available.

**Results:**

Disease prediction worked best for diseases with specific treatment options (e.g., diabetes). Random forests achieved the best performance in 56% of all classification problems. For 25 diseases, drug utilization by ATC chapter was the most important feature in the prediction. Prevalence rates predicted for the full population were close to those reported previously for few diseases only, and showed large deviations for other diseases.

**Conclusions:**

Information on health care utilization from claims data may be used to predict the presence of diseases, but predictive performance varies across diseases, warranting further research on population-wide disease prevalence rates with incomplete information on diagnostic data.

**Supplementary Information:**

The online version contains supplementary material available at 10.1186/s13690-025-01813-y.

**Table:** 

Text box 1. Contributions to literature	
- Diagnostic coding is often missing in administrative billing data, especially in outpatient care. - This paper contributes to the literature by applying machine learning algorithms to predict the probability of having any of 32 diseases in a population of hospitalized patients in Switzerland. - Variable importance analysis shows that prescribed drugs as well as other healthcare services such as specific physician services indicate the presence of a disease. - Our disease prediction models can be used to estimate prevalences in the full population.	

## Background

Health care systems generate large amounts of data about patients’ health status, health care utilization, and health care spending. Morbidity measures are used in epidemiological research to monitor the burden of diseases or in risk equalization schemes to compensate insurers for their populations’ elevated risk of becoming ill. They are also used together with spending data to assess the economic burden of diseases in cost-of-illness studies. The sources of information are usually large surveys or administrative routine data [1], mandatory registries [2], or insurance claims data [3, 4]. However, despite the large amount of data generated, a comprehensive assessment of the morbidity based on routinely collected administrative data is still challenging in many health care systems due to missing diagnostic information, especially in outpatient care.

The availability of diagnostic data in administrative data varies significantly across different health care systems. Some public, tax-funded systems such as the ones in Northern European countries collect comprehensive diagnostic data in population-based registries [5–7]. Such information is usually less easily available in decentralized systems. In Switzerland, for example, a multitude of payers collect data on service provision for billing reasons, but almost no diagnostic information. There is currently no diagnostic coding by providers of outpatient care. This contrasts with the inpatient sector, where each case is routinely registered in a mandatory nation-wide inpatient hospital registry (HospReg; Medizinische Statistik der Krankenhäuser) [8] using detailed coding based on the international classification of diseases (German modification), version 10 (ICD-10-GM) for diagnoses and the Swiss surgical procedure classification (CHOP) for treatments.

Claims data from insurers in the Swiss mandatory health insurance (MHI) are collected for billing reasons and hold detailed and comprehensive information about health care services and spending at the individual level. Inpatient diagnostic and procedure data are not transferred to the insurer, as they are not relevant to billing^1^ (i.e., insurers know only the costs derived from the diagnosis-related group (DRG) weight). In health care systems without comprehensive diagnostic coding in all sectors, deriving substitutes for diagnostic codes from routinely collected administrative data containing information on services may be an economical alternative to fill this gap.

Obtaining morbidity information for the (insured) population may be of relevance for various reasons such as epidemiological or health services research. One example is the estimation of the prevalence of specific diseases as well as differences in the prevalence across different populations (e.g., from different regions within a country or different health insurers). Previous research that required morbidity indicators for insured individuals used measures based on drug utilization from claims data (e.g. [3, 9]). A recent cost-of-illness study for Switzerland complemented the pharmacy-related information with other ‘diagnostic clues’ from claims data, i.e., disease-specific procedures and treatments that can be identified based on billing positions in fee-for-service catalogues [4]. The identification of these clues was based on theory and prior clinical knowledge. However, it remained unclear to what extent additional information from claims data could be useful in the disease identification and prediction of prevalence rates.

Statistical learning methods like machine learning refrain from making a priori assumptions about the relevance of single features for prediction. Previous research has pointed out the potential benefits of using machine learning methods for research in population health, especially when applied to linked data sets [10]. Machine learning applications in health care include the prediction of the best treatment plan for patients with breast cancer [11], the prediction of complications after stroke [12], the optimization of digital contact tracing [13], or the identification of cancer from mammograms [14]. Only few existing studies used claims and/or prescription data to predict the presence of single diseases. Examples are the prediction of asthmatic patients based on medical claims or prescriptions [15], the prediction of incident diabetes patients using information about past reimbursed services [16], the prediction of incident Parkinson’s patients in Medicare claims data using the elastic net algorithm [17], and the prediction of previously undiagnosed patients with non-tuberculous mycobacterial pulmonary disease in Germany based on drugs and procedure codes [18]. Many existing studies aimed to predict the presence of a disease in undiagnosed patients or to identify patients before the disease onset (i.e., patients at risk, e.g., of having diabetes [19]). Closely related to our work, Slobbe et al. (2019) used detailed data on drug use in the Netherlands to find the most important predictors for the presence of 29 chronic diseases in claims data and to infer prevalence rates from the individual-level probabilities of having a disease [20].

Previous studies for Switzerland have used machine learning methods together with claims data from the MHI to predict changes in individual health care spending [21] or aggregate health care spending over time [22]. To the best of our knowledge, claims data from MHI have not yet been used to predict the presence of a disease applying a data-driven approach.

The goals of this study are threefold. *First*, we aim to train a set of machine learning models to predict 32 single diseases in inpatient care from the billed services in outpatient care. To this end, we link individual-level claims data from SWICA, a major insurer in MHI in Switzerland, in 2017 with the inpatient case-level data containing ICD-10-GM codes. This creates a unique dataset. *Second*, we aim to identify the most relevant predictive features using importance measures and to compare them with the set of more theory-based clues used in previous research. *Third*, we select diseases with sufficient prediction performance and apply the model trained on the hospitalized population to predict diseases in the non-hospitalized population for which no diagnostic coding was available. These predictions are used to obtain prevalence estimates for the full population.

Our study contributes to the literature in several ways. *First*, it demonstrates the benefits of using a variety of measures of health care utilization (e.g., specific physician services) in disease prediction. Previous research has predominantly focused only on drug utilization as disease predictor. *Second*, it shows that assessing several algorithms improves the quality of the predictions. *Third*, it shows for which diseases predictions based solely on health care use are sufficiently precise to allow for the application of data-driven approaches to estimate disease prevalence in the general population for whom no diagnostic coding is available. *Fourth*, it shows which features are the most important ones in the prediction of specific diseases and for which diseases these correspond to the ones used in a theory-based approach. In cases where the features differ, the findings may help generate new hypotheses about the relationship between health service consumption and diagnoses.

## Data

We used two data sources and linked them. *First*, anonymized insurance claims data at the individual level from SWICA health insurance. *Second*, anonymized case-level data from the Swiss hospital inpatient registry HospReg.

The anonymized claims data set includes a random sample of 709,788 individuals enrolled with SWICA, a major provider of MHI with a market share of around 10%.^2^ For each insured individual, it contains total gross spending (i.e., including co-payments and deductibles) in MHI in 2017 by provider type (e.g., hospital, neurologist, laboratory), total gross spending covered during maternity, total aggregated spending by chapter or subchapter of national fee-for-service tariff catalogues, as well as the number of packages and the aggregated spending by the therapeutic/pharmacological subgroups of the Anatomical Therapeutic Chemical (ATC) classification of the World Health Organization for all drugs covered by MHI (based on a positive list (Spezialitätenliste; SL)). In addition, it contains the sex and age by 5-year bins for each individual, but no other personal information such as domicile or socioeconomic variables. All utilization data were aggregated at the level of tariff catalogue chapters, i.e., the data does not include single billing positions. This is because of the trade-off between using predictors as specific and with as much information as possible and generating an adequate number of predictors that can still be handled in the modelling. By aggregating and thereby reducing the several thousand billing positions from the tariff catalogues, we lost some information but ended up with a reasonable number of features.

We included utilization data of services from the following fee-for-service tariff catalogues: TARMED (tarif médical; diagnostic procedures and treatments by physicians), list of laboratory tests (Analysenliste; AL), and list of therapeutic medical devices (Mittel- und Gegenständeliste; MiGel). The single billing codes were grouped into chapters and subchapters within each catalogue. One example is the radiology chapter in TARMED, which contains the five subchapters X-ray, computerized tomography (CT), Magnetic Resonance Imaging (MRI), ultrasound, and angiography. The aggregation at the level of chapters did not require any clinical knowledge but was entirely driven by the structure of the tariff catalogues.

Table 1 summarizes the health care utilization information in the claims data including the level of disaggregation and examples of the structure.

**Table 1:** Health care utilization information in claims data

Information/Tariff catalogue	Services covered	Tariff catalogues: Number of codes	Number of chapters and/or subchapters available (used)	Example of chapter or subchapter used
Gross spending by physician specialization	Specialized physician services	-	19 (19)	Neurology
Gross maternity spending	All	-	1	-
TARMED (tarif médical)	All physician services	Approx. 4600	55 (52)	X-rays (chapter 39.02)
Analysenliste AL	Laboratory tests	Approx. 1800	15 (15)	Cytogenetics (chapter 2.2.1)
Mittel- und Gegenständeliste MiGel	Therapeutic devices	Approx. 700	20 (20)	Hearing aids (chapter 13)
Spezialitätenliste SL	Drugs covered by MHI	Approx. 9700	223 (208)	Insulins and analogues (ATC A10A)

Table summarizes the health care utilization measures from claims data; the number in parentheses in column 4 refers to the number of (sub) chapters used in the modelling (selected if at least 100 individuals had positive utilization)

HospReg is routinely published by the Federal Statistical Office (FSO) and contains records of all inpatient care episodes in Swiss hospitals since 1998. The full HospReg contains demographic information about the patient as well as episode-specific information such as procedure codes, mode of admission and discharge, length of stay, as well as one main and up to 49 secondary diagnoses coded according to ICD-10-GM. Individuals can be tracked across time and hospitals via an anonymous identifier. We discarded all the variables from HospReg except for the anonymized patient ID and the diagnostic information. We used all diagnostic codes (i.e., main and secondary diagnoses) of all episodes in 2016 and 2017 of all individuals who were hospitalized at least once in 2017, i.e., the year for which we have the complete record of outpatient utilization data. We thereby assumed that diagnoses from 2016 were still present in 2017. As this assumption does not hold for more acute conditions (e.g. most infectious diseases), we excluded some diseases from the list of predicted conditions.

## Methods

### Data linkage

In collaboration with the FSO, we developed a procedure to identify the patients insured with SWICA in the HospReg data. Both the FSO and SWICA created patient identifiers based on individual information (e.g., date of birth), which were then translated randomly into anonymized identifiers by the FSO before the record linkage. The linkage of the utilization and diagnostic data was performed by the authors, thereby avoiding that neither the FSO nor SWICA had access to the other party’s data. This process maintained the anonymization of patient’s records.

As one patient represented one observation in the claims data but potentially multiple observations in HospReg, we aggregated all the diagnostic codes from all stays in 2016 and 2017 at the patient level. This allowed for linkage of the two data sets at the patient level.

Linkage of HospReg with other routinely collected data is not done on a regular basis. To the best of our knowledge, only one previous study linked HospReg data with individual claims data. Halfon et al. (2013) checked how diagnoses from HospReg corresponded to prescriptions of disease-specific drugs, captured by ATC codes, using data from 2005 and 2006 [23].

### Classification of diseases

We classified diseases according to the classification used in the Global Burden of Disease (GBD) project [24]. The original classification contains 359 diseases and injuries which can be grouped at four different levels of aggregation. We adjusted the original classification to identify a comprehensive set of 32 mutually exclusive diseases (corresponding roughly to GBD level 3) in 14 major disease categories (corresponding to GBD level 2). All residual diseases within a disease category (e.g., “other cardiovascular diseases”) were excluded as they do not represent clinically interpretable conditions. We used the mapping of ICD-10 codes to the GBD diseases as provided by the GBD project and adjusted it to match the ICD-10-GM classification used in Switzerland. The same disease classification was used in previous research for Switzerland [4].

### Data-driven disease identification

The main goal of this study was to solve 32 binary classification tasks, one for each disease. We used detailed health care utilization information (see Table 1) as features, i.e., explanatory variables, in the models. We captured utilization in terms of spending for each type of feature. In addition, we included age (in 5-year groups) and sex of the individual as explanatory variables. A complete list of all features used in training can be found in the supplementary material (additional file 1).

The total number of potential features was 317. Including such a high number of predictors in the modelling significantly affects computation speed. We therefore applied a feature selection based on the Boruta algorithm to select the most relevant features for the model training [25].

We evaluated and compared two types of tree-based ensemble machine learning classifiers: Random Forest (RF) and Gradient Boosted Machines (GBM). RF has been used in similar research aiming at the identification of patients with a certain disease [20, 26, 27]. GBM has outperformed RF in a recent study aiming at predicting diabetes in Canadian patients [19]. We used 70% of the linked data in training and 30% in testing. The training dataset was used to train the model, while the test dataset was used to evaluate how well the trained model performed on unseen data. Furthermore, we applied a grid search to tune hyperparameters, using fivefold cross-validation. The best combination of hyperparameters was determined based on the maximized area under the precision-recall curve (AUCPR). We estimated the final model using the full training data set and evaluated it in the unseen test data. The hyperparameters of interest in the RF were the number of features randomly selected at each split in the trees, the minimum node size, and the share of observations that are sampled for each tree. For GBM, we tuned the number of trees, the learning rate (shrinkage parameter), the interaction depth, as well as the share of observations sampled for each tree. Details about the grid search are provided in supplementary material 2.

Each of the 32 classification problems was characterized by a class imbalance, i.e., there were far less individuals with the disease than people without it. Previous studies which predicted disease probabilities made several adjustments to the algorithms to account for this imbalance [27]. There are different ways to address this problem, either by adjusting the class distribution in the data preparation stage before training (e.g., under- or over-sampling such as the synthetic minority over-sampling technique (SMOTE) [28]) or in the training process itself. We refrained from applying any adjustments to the class imbalance in the data preparation stage, as recent research has shown that class imbalance corrections in machine learning applications in health care can cause model mis-calibration [29]. In the training process, we evaluated cost-sensitive learning in the RF models, which allows for a higher weight of observations of the minority class in the optimization.

The two algorithms combined with the cost-sensitive version of the RF algorithm resulted in three models for each disease. We selected the best model for each disease based on the highest AUCPR score. Using the best model, we then predicted individual-level probabilities/scores of being a prevalent case. No conversion to hard labels was made.

In order to obtain comparable variable importance measures for each classification problem, we used the feature importance from RFs (R ranger package [30]) for all diseases. Importance refers to the Gini index for classification and is defined as the mean decrease in impurity. A higher value means a more important feature for the prediction.

### Disease prevalence estimation in non-hospitalized population

All the features used in the prediction of diseases were also available for those who were not hospitalized in 2017 and thus not included in HospReg. We had no diagnostic information (labels) for these individuals. We applied the models for the hospitalized population (with labels) to predict the probability of diseases at the individual level in the non-hospitalized population (without labels). We added the data-driven predictions (i.e., the individual disease probabilities) for the non-hospitalized to the observed prevalence based on the HospReg coding (ICD-10-GM codes) for the hospitalized population and averaged the predictions to obtain an overall prevalence rate for the full insured population. This is similar to a Dutch study which computed regional prevalences from individual predicted disease probabilities [31]. We weighted the individual predictions using sex and age specific weights to account for the slightly different structure of the sample and the general Swiss population. We compared the prevalence rates from the data-driven approach to the clues-based estimates from previous research [4] and to the GBD estimates for Switzerland [32].

## Results

### Data-driven disease identification

The sample of those who were hospitalized at least once in 2017 consisted of 70,571 individuals. This corresponded to 9.9% of the full sample of all insured, meaning that 639,217 individuals (90.1%) were not hospitalized in 2017.^3^

For each of the 32 diseases, we chose the best machine learning model according to its AUCPR score. Table 2 lists all diseases along with the number of features selected for the modelling, the best model with the associated AUCPR score, as well as the estimated prevalence from the best model and the observed prevalence in the test data.

**Table 2:** Best machine learning algorithm selected for each disease

Disease category (GBD level 2)	Disease (GBD level 3)	Number of features selected	Best model	PR AUC of best model	Estimated prevalence of best model (= mean probability score; %)	Observed prevalence (%)
Communicable diseases						
	HIV, AIDS	11	RF WGT	0.19	0.05	0.07
	Hepatitis	52	GBM	0.08	0.35	0.44
Maternal and neonatal disorders	Maternal and neonatal disorders	109	GBM	0.72	10.61	10.77
Nutritional deficiencies	Nutritional deficiencies	165	GBM	0.46	10.45	10.59
Neoplasms						
	Colon and rectum cancer	63	RF	0.36	1.56	1.43
	Trachea, bronchus and lung cancer	50	GBM	0.27	0.98	0.82
	Breast cancer	72	RF WGT	0.74	1.75	1.66
	Prostate cancer	50	RF	0.57	1.21	1.12
Cardiovascular diseases						
	Ischemic heart disease	103	RF	0.70	9.55	9.32
	Stroke	128	RF	0.53	8.29	8.03
	Hypertensive heart disease	78	GBM	0.42	5.91	5.97
	Atrial fibrillation and flutter	82	GBM	0.65	6.74	6.43
Chronic respiratory diseases						
	Chronic obstructive pulmonary disease (COPD)	69	GBM	0.55	3.54	3.44
	Asthma	48	RF	0.29	1.93	1.76
Digestive diseases						
	Cirrhosis and chronic liver disease	84	RF WGT	0.20	2.13	1.98
Neurological disorders						
	Alzheimer’s and dementia	65	RF WGT	0.47	2.54	2.52
	Parkinson’s disease	39	RF	0.72	0.74	0.70
	Epilepsy	84	RF	0.48	1.89	1.64
	Multiple Sclerosis	33	RF	0.34	0.30	0.36
Mental and substance use disorders						
	Schizophrenia	46	RF	0.53	0.81	0.82
	Depression	94	RF WGT	0.53	7.01	6.86
	Attention Deficit Hyperactivity Disorder (ADHD)	47	RF WGT	0.30	0.46	0.42
	Alcohol and drug use disorders	112	RF WGT	0.43	5.37	5.13
Diabetes and kidney disease						
	Diabetes mellitus	77	GBM	0.82	7.88	7.77
	Chronic kidney disease	141	GBM	0.58	8.32	8.26
Skin and subcutaneous diseases	Skin and subcutaneous diseases	137	GBM	0.27	6.24	6.40
Sense organ diseases	Sense organ diseases	113	RF	0.32	5.64	5.30
Musculoskeletal disorders						
	Rheumatoid arthritis	18	RF WGT	0.08	0.22	0.19
	Osteoarthritis	84	GBM	0.63	5.74	5.76
	Low back pain	63	GBM	0.15	2.39	2.48
	Osteoporosis	68	GBM	0.42	3.43	3.30
Other non-communicable diseases						
	Oral disorders	53	GBM	0.09	0.89	0.88

*GBD* Global Burden of Disease, *GBM* Gradient boosted machines, *RF* Random Forest, *WGT* weighted (cost-sensitive learning), *PR AUC* Area under the Precision-Recall curve

prevalence based on ICD-10-GM codes from HospReg in 2016 and 2017

The boosting algorithm performed best. In 14 out of 32 classification problems (44%) the GBM algorithm yielded the highest AUCPR, followed by the standard RF (n = 10; 31%) and the cost-sensitive (“weighted”) RF (*n* = 8; 25%).

AUCPR was below the value expected from a random classifier (with a baseline equal to the prevalence of the disease) for two diseases: hepatitis and rheumatoid arthritis. These diseases were excluded from any further analyses.

In 10 out of 32 diseases (31%), the predicted prevalence rate from the best model (mean probability) was lower than the observed prevalence in the test data, in 22 diseases (69%) it was higher.

### Variable importance

**Fig. 1:**
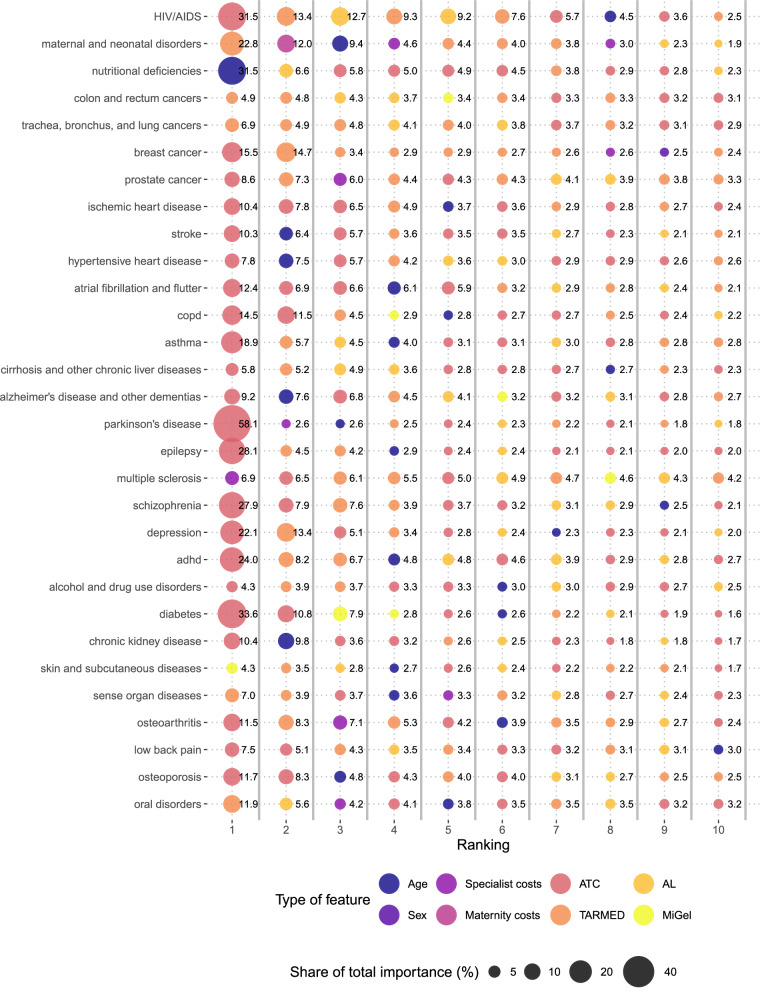
Variable importance from RF models (proportion of 10 most important variables of total importance by disease, %). Figure shows the variable importance (obtained from RF models) for the ten most important features in the prediction of each disease, by type of feature. The size of the circle refers to the share of total importance, the colour refers to the type of feature. ATC: Anatomical therapeutic chemical; AL: Analysenliste (laboratory tests); MiGel: Mittel- und Gegenständeliste (list of medical devices); TARMED (physician services)

Figure 1 shows the ten most important features in the disease prediction, ranked from 1 (left) to 10 (right). The size of each circle and the number next to it refer to the feature’s proportion of total importance. The colour of each circle indicates the type of feature. For example, dark blue refers to age, purple refers to sex.

In 25 diseases, spending for drugs by ATC chapter was the most important predictor. In 26 cases, age was among the ten most important features. The share of total importance explained by the top features was especially high in Parkinson’s disease (ATC N04B (dopaminergic agents); 58%), diabetes (ATC A10B (Blood glucose lowering drugs, excluding insulins); 34%), HIV/AIDS (ATC J05A (Antivirals); 32%), nutritional deficiencies (Age; 32%), and maternal and neonatal disorders (TARMED 22.02 (Diagnostics and therapy of the female genital organs); 23%). All types of features appeared at least once among the top 10 features of all diseases.

The complete list of the ten most important features for each disease including their names and their proportions of the total importance can be found in the supplementary material (additional file 3).

Finally, we computed the importance share by type of feature and disease. The mean share of drugs/ATC was highest (49.7%), followed by TARMED (27.8%), AL (8.7%), costs by specialist type (5.7%), and age (5.1%). The other feature types showed shares below 5%.

We checked if the most important features in the data-driven disease prediction were the same as in a more conventional, theory-driven and ‘clues-based’ identification approach followed in previous research for Switzerland [4]. Stucki et al. used theory-based measures of utilization (e.g., drugs used in diabetes treatment) to identify diseases in claims data. The data-driven strategy may indicate if there are other relevant clues to complement theory-based ones. Table 3 shows the five most important variables for the 14 diseases for which the importance of the top 10 predictors summed up to at least 50% of the total importance. Variables in bold letters were either used in the exact same way as a disease-specific clue in previous research (e.g., ATC chapter N04B for Parkinson’s) or contained single billing positions that were used as clues (e.g., ATC code N06BA04 within N06B for ADHD).

**Table 3:** Top 5 features according to variable importance for selected diseases

Disease	Rank 1	Rank 2	Rank 3	Rank 4	Rank 5
HIV/AIDS	**ATC J05A**	TM 00	AL 3.2	TM 39.01	AL 4.2
Maternal and neonatal disorders	TM 22.02	**Maternity**	Age	**Gynaecology**	TM 03
Breast cancer	**ATC L02B**	**TM 23**	TM 37	TM 22.01	TM 32
Prostate cancer	**ATC L02A**	TM 21	Urology	TM 00	**ATC L02B**
Atrial fibrillation and flutter	ATC B01A	ATC C07A	**ATC C01B**	Age	ATC C03C
Asthma	**ATC R03A**	TM 00	AL 1.2	Age	**ATC R03B**
Parkinson’s disease	**ATC N04B**	Neurology	Age	TM 00	ATC N06D
Epilepsy	**ATC N03A**	**TM 05.01**	TM 00	Age	ATC N06A
Multiple Sclerosis	Neurology	ATC M03B	TM 05.01	TM 00	ATC N02B
Schizophrenia	**ATC N05A**	ATC N04A	TM 02	TM 00	ATC N05B
Depression	**ATC N06A**	TM 02	ATC N05A	TM 00	ATC N05B
ADHD	**ATC L02B**	TM 02	TM 00	Age	AL 1.2
Diabetes	**ATC A10B**	**ATC A10A**	**MiGel 21**	MiGel 3	ATC C10A
Osteoarthritis	ATC B01A	TM 39.02	Orthopaedics	TM 24.01	**ATC M01A**

Table shows the top 5 features in the disease prediction of diseases in which the top 10 features had a share of at least 50% of the total variable importance. Rheumatoid Arthritis was excluded due to low model performance. Features in bold have been used in previous research using theory-based disease identification. The letters stand for the type of feature (e.g., Anatomical therapeutic chemical (ATC) for drugs), the numbers refer to the (sub)chapter of the tariff catalogue; MiGel: Mittel- und Gegenständeliste (list of medical devices; e.g., MiGel 21 “measurement systems for body conditions and functions”); TM: TARMED (physician services; e.g., TM 39.02 X-Ray); AL: Analysenliste (laboratory tests; e.g., 3.2 virology)

For all diseases except multiple sclerosis, at least one of the five most important features agreed with the theory-based clues. The most important features in the prediction of ten diseases (e.g., Parkinson’s disease (ATC N04B) and diabetes (ATC A10B)) were from the list of drugs and were also used in the theory-based disease identification. Age was an important predictor for six diseases but could not be considered in theory-based approaches. Even though TARMED chapter 00 (“Basic physician services”) contains mostly general procedures, it was an important predictor for many diseases. This might reflect a general increased demand for health care services by patients with that disease, as this chapter contains a billing position that is used every time a patient has an outpatient physician contact (GPs and specialists in practice or hospital).

### Disease prevalence estimation in non-hospitalized population

**Fig. 2:**
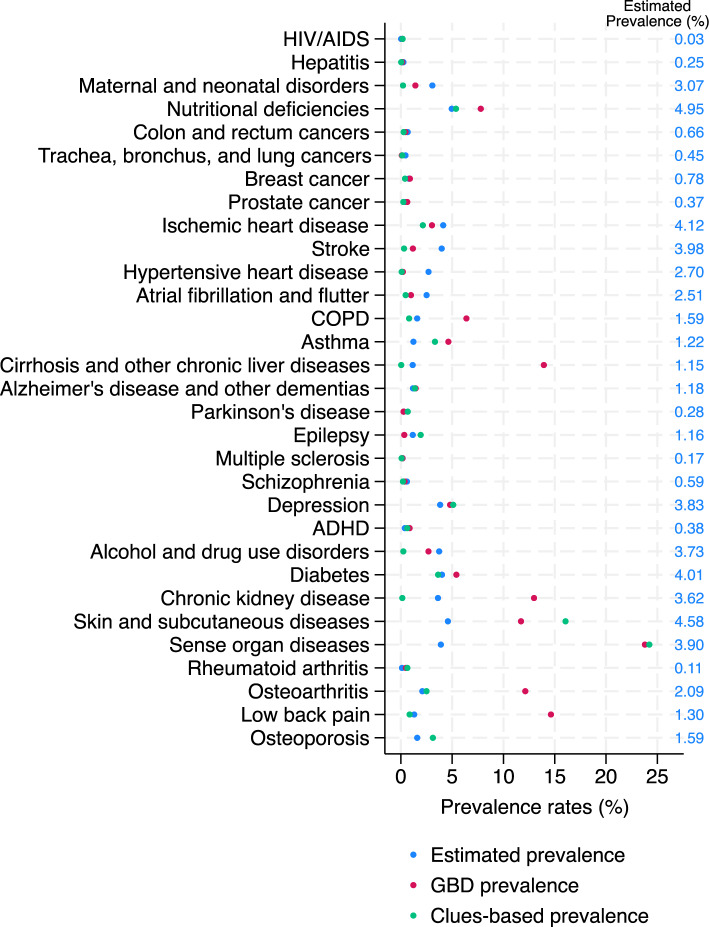
Comparison of estimated prevalence with results from the Global Burden of Disease (GBD) study and a clues-based/theory-based disease identification. Figure shows the estimated prevalence (year 2017; blue dots) and results from the GBD study 2023 (year 2017; red dots) and from the clues-based disease identification in Stucki et al. 2021 (year 2017; green dots). The numbers in blue refer to the estimated prevalence (blue dots)

Figure 2 reports the estimates for the prevalence in the full population, based on the combination of observed prevalences in the hospitalized population and individual predicted probabilities for the non-hospitalized population derived from the best models according to Table 2 (blue dots and blue numbers). The red dots refer to estimates from the GBD study 2023 for Switzerland [32], and the green dots refer to the prevalence estimated based on theory-based clues [4]. All estimates are for 2017. The estimated prevalence was in many cases higher than the clues-based prevalence, but lower than the one according to the GBD (e.g., COPD or chronic kidney disease). In all cardiovascular diseases (ischemic heart disease, stroke, hypertensive heart disease, atrial fibrillation and flutter), the estimated prevalence was higher than both the GBD and the clues-based result. The three estimates were very close for diabetes and depression.

## Discussion

### Summary and contributions

We used individual-level claims data from a major Swiss health insurer and diagnostic data for hospitalized patients from the nation-wide inpatient registry to assess performance of RF and boosting algorithms in the prediction of 32 diseases in hospitalized patients. We found that spending by ATC chapter was in many cases an important predictor of a disease, but other types of predictors (e.g., type of physician services or the patient’s age) were also among the most important ones. The most important features corresponded well to the ones used previously in theory-based disease identification.

Our study aimed to demonstrate a process of deriving informed guesses for diagnoses from the rich data that is available in health insurance claims data. Our main contribution is to highlight the benefits of using detailed information on health care utilization in a broad range of service types available from claims data to describe the morbidity in the insured population. Information about the use of non-pharmacological health services is in several cases a useful predictor for the presence of a disease. This broadens the view of previous similar research that focused mainly on using drug utilization as predictors.

If the prediction models yielded a sufficiently high predictive power for diseases which are difficult to identify based on simple clues such as disease-specific medication, prevalence estimation would be substantially facilitated. We show that evaluating different classifiers and choosing the best among them improves the predictions. The approach followed in this paper may be interesting for applications in similar countries with limited diagnostic information in administrative data, even if the available features are likely to be different in other health care systems.

Our methodology involves the operationalization of health care utilization (e.g., by aggregating utilization by chapters of tariff catalogues), the selection of relevant features to reduce the complexity of the prediction problem, the training and choice of the appropriate or best model, as well as the interpretation of the predictions resulting from this model. We consider three cases in which data-driven predictions may be useful. *First*, when there are no single unique ‘clues’, but a (possibly complex) combination thereof that indicates the presence of a disease. *Second*, to generate hypotheses about links between observed health care utilization and disease prevalence. *Third*, in cases where there is suspected under-coding of diseases. This is particularly relevant in our study, as not all diseases are coded in HospReg. Detecting the utilization patterns of the prevalent patients with a coded diagnosis may help to identify additional prevalent patients that did not have the corresponding diagnostic code.

Our approach has one important advantage compared to more established ways of identifying diseases based on routinely collected billing data. Instead of making a priori assumptions about which services indicate the presence of a disease, comprehensive measures of outpatient service utilization were used as features in machine learning models with the goal to predict the disease. A pure theory-driven disease identification may be problematic in cases in which treatment patterns are beyond clinical theory [20] or not standardized (e.g., by clear guidelines). Furthermore, some services may be used for the treatment of multiple diseases, and only interactions with other services may indicate the underlying health condition. Examples are antithrombotic agents, immunosuppressant drugs or opioids.

### Interpretation and comparison with literature

#### Model performance

For many of the 32 diseases included in our analysis, the machine learning classifiers performed substantially better than a random classifier, as demonstrated by the AUCPR values. However, the performance varied. While diseases such as diabetes, breast cancer and Parkinson’s disease achieved very high AUCPR, other diseases such as cirrhosis or oral disorders had low AUCPR. The latter seem to have a low (recorded) prevalence in the inpatient setting, which may also explain the difficulty of the algorithm to identify cases from the available data. However, the predicted prevalence and observed prevalence in the test data usually corresponded well. In more than two thirds of all diseases, the predicted prevalence was slightly higher than the observed one. This might indicate under-coding of diseases in HospReg. As the coding guidelines state that only diagnoses that are associated with the use of resources in the hospital are to be coded, not all diseases a patient suffers from are coded in the hospital inpatient setting (even if treated in other settings).

#### Variable importance

In the variable importance analysis, we showed that among the most important predictors for each disease, there were many drugs. Drug use by ATC 4-digits chapters was the most important predictor for all but seven diseases. In many cases, these codes were also used in theory-based disease identification from claims data in previous research [4] (e.g., ATC R03A in asthma and J05A in HIV/AIDS). A comparison is interesting, but it should be noted that a theory-based approach need not be correct, e.g., when drugs are used in the treatment of multiple diseases, e.g., immunosuppressants (ATC L04A) or opioids (N02A). Machine learning models do generally not allow for causal interpretation. However, the importance analysis can help to generate new hypotheses about possible causal links between measures of healthcare utilization and the existence of specific diseases, which can be assessed and validated by clinical experts and further investigated in subsequent studies.

The study by Slobbe et al. (2019) estimated individual probabilities of having one of 29 chronic diseases based on aggregated medication data (the same ATC 4-digits classification as in our study) for almost 300,000 patients in the Netherlands using RF [20]. The authors aimed to show for which diseases machine learning algorithms can be used for prevalence estimation, and which drugs should be used as features. One major finding of their study was that disease-specific drugs, as suggested by theory (e.g., diabetes medication), were among the most important predictors in 16 of 17 diseases. Our results are similar, even if we included a much broader range of features than just drugs by ATC chapter. Our ranking of the most important medication variables (i.e., 4-digit ATC chapters) corresponded very well to the Dutch study. In all the 15 diseases that were included in both studies, there was at least one common feature among the top three predictors. In 10 of these diseases, the most important feature was the same (e.g., ATC R03A in both asthma and COPD or ATC N05A in schizophrenia). The three most important predictors for Alzheimer’s and other dementias were exactly the same in both studies (age and ATC chapters N05A and N06D). Others shared two common top features, e.g., depression (ATC chapters N06A and N05A), Diabetes (ATC chapters A10B and A10A) or osteoporosis (ATC chapters A12A and M05B). However, the fact that other features than ATCs were among the most important predictors shows that healthcare services other than drugs may indicate the presence of a disease, including physician services as recorded in the tariff system or medical device use. This is an important and novel insight from this paper.

We were also able to confirm the finding by Slobbe et al. (2019) that a good correspondence between algorithm-based and theory-based predictors did not necessarily mean that model performance was sufficient. The authors concluded that predictions were good for only four out of 29 diseases (Parkinson’s disease, diabetes, osteoporosis, and heart failure).

Halfon et al. (2013) checked the correspondence of drugs-based morbidity measures and diagnoses using the same kind of linked data, i.e., HospReg and claims data [23]. They showed that many diseases showed a poor fit between the drugs-based and diagnoses-based measures. We were able to partly confirm this result, as not all substances used in the theory-based identification appeared as top predictors in the data-driven approach.

#### Disease prevalence estimation

We obtained prevalence estimates in the full population for all diseases and compared the results to two other sources. However, we were not able to conclude if the resulting prevalence estimates were reflecting the true prevalence. Comparing the prevalence to previous literature using similar claims data and the GDB estimates, we think that the data-driven approach yielded realistic estimates for cancers, diabetes and depression. The model-based prevalence estimate was generally higher than the other estimates for cardiovascular diseases. The prevalences of chronic respiratory diseases seem to be underestimated (COPD 1.59% and asthma 1.22%). The model-derived prevalence rate for diabetes (4.01%) corresponded well with the theory-based estimate using claims data (3.63%). This condition is easily identified using medication claims data and the benefit of using more predictors is small. The big difference between the GBD estimate for chronic kidney disease (12.98%) and our estimate (3.62%) shows that the result depends substantially on what we want/can measure; while the GBD estimates comprise prevalent patients of all stages, we were likely to capture only treated patients in more severe stages. Moreover, the GBD estimates refer to overall disease prevalence, while our data contains mostly treated patients. A combination of the theory-based and the data-driven approach could help identify prevalent individuals with the typical treatments and those with alternative treatment patterns.

### Limitations and future research

This study has several limitations.

*First*, we trained our disease prediction models on hospitalized patients only, as diagnostic codes were not available for the non-hospitalized. While the predictors are available for all hospitalized and non-hospitalized individuals, the associations between a disease and the health care utilization measures might, however, differ between the two groups, as hospitalized patients are likely to be more severely ill. This may cause a bias in the disease probability prediction and lead to an over- or under-estimation of the disease prevalence. Due to data limitations, it was not possible to analyze the generalizability of the machine learning models for the full population.

*Second*, not all prevalent patients are labelled in HospReg data, as only diseases which affect the inpatient treatment need to be coded. Conditions that are mainly treated in outpatient settings are not coded in inpatient care. Further sources of under-coding in administrative data include incomplete clinical documentation, the focus on primary and secondary diagnoses relevant for billing, and variations in coding resources and practices across hospitals. Therefore, correct diagnostic coding for HospReg might still result in mis- or under-coding for our analyses. We partly took this limitation into account by including all diagnostic data available from all hospitalizations in 2016 and 2017 for hospitalized patients in 2017. However, we cannot rule out that such data limitations lead to biases in our prevalence estimates.

This approach comes with the *third* limitation, as some chronic conditions might still not be captured, even if they were coded in previous inpatient stays before 2016.

*Fourth*, we only used outpatient utilization data from one year. It is possible that some patients used disease-specific outpatient services more extensively *before* being admitted to the hospital for that reason. This relationship can only be revealed if observed in the same year; if patients consumed the services in the year before the inpatient stay, we did not observe it in the data.

*Finally*, some drugs might be prescribed exclusively in inpatient settings. In such cases, we might not identify all disease-specific predictors, simply because we do not observe them in our set of features (e.g., highly specialized drugs for cancer [23]).

Future research should aim to include features at a more granular level (e.g., specific substances instead of ATC chapters). This would likely improve model performance, but also comes at higher computation times. In our study, we wanted to include a broad range of predictors from most outpatient service categories, which is why we chose to use more aggregated measures. Another area for future research would be to use a different source for the disease labels; electronic medical records with complete diagnostic coding are currently not available in Switzerland but might be in the future and might then provide a more complete data source for the whole population.

### Policy implications

Health policy is often struggling to gather all information required to define effective policy measures, be it for cost containment or for the prevention and treatment of (chronic) illnesses. Many of these data limitations and gaps cannot be easily fixed. In Switzerland, there is currently no systematic diagnostic coding in outpatient care. However, by using existing administrative and routinely collected data and exploiting their extra benefits after linkage, some of the limitations can potentially be overcome. Our machine learning framework for the prediction of diseases at the individual level and the aggregation for prevalence estimations could help better describe morbidity in the population. This is especially important for public health policy such that scarce resources can be allocated efficiently. Using data-driven disease prediction models could also prove useful for sub-group analyses, e.g., by age or geographic region, for which often no epidemiological data is available. This was the approach followed in a recent study for the Netherlands [31].

## Conclusion

This study used detailed claims data of the insured population of a major insurer in the Swiss MHI and linked it to the data from inpatient care to obtain disease labels for the subset of hospitalized patients. We used machine learning techniques to solve binary classification problems for 32 diseases, using measures of outpatient care utilization, age, and sex as predictors. Several disease prediction models showed high values of AUCPR, indicating the potential value of using machine learning algorithms in disease prediction. The trained models were used to obtain prevalence estimates for the full population. Variable importance analysis revealed interesting insights into outpatient care utilization patterns of patients with a certain disease. These findings may help generate new hypotheses about the relationship between treatments and diseases.

## Supplementary Information

Additional file 1. Features from tariff catalogues used for training.

Additional file 2. Details about hyperparameter tuning.

Additional file 3. Variable importance.

## Data Availability

The claims data are confidential and not available. The HospReg data can be obtained from the Federal Statistical Office (gesundheit_dsv@bfs.admin.ch) after filing an application and signing a data use and protection agreement.

## Abbreviations

ADHD: Attention Deficit Hyperactivity Disorder
AL: Analysenliste (list of laboratory tests paid by MHI)
ATC: Anatomical-therapeutic chemical
AUCPR: Area under the precision-recall curve
COPD: Chronic obstructive pulmonary disease
DRG: Diagnosis-related group
FSO: Federal Statistical Office
GBD: Global Burden of Disease
GBM: Gradient Boosted Machine
ICD-10(-GM): International Classification of Diseases, 10th version, (German Modification)
MHI: Mandatory health insurance
MiGel: Mittel- und Gegenständeliste (list of medical devices paid by MHI)
RF: Random Forest
TARMED: Tarif médical (outpatient tariff system for physician services)

## Footnotes

1 Insurers only get access to information about diagnoses and medical procedures if needed to check if the treatments were appropriate and cost-effective. In such cases, they receive a Minimal Clinical Dataset which contains ICD-10-GM diagnoses and CHOP procedure codes.

2 The sample is similar to the Swiss population with respect to the age/sex structure and spending per capita. It consists of slightly fewer elderly people and the spending per capita is lower than in the general population [4].

3 There were 6,718 individuals who were included in HospReg, but did not have any hospital costs according to the claims data, and 8,749 individuals who had positive inpatient care costs but were not linked to HospReg. It was not possible to determine the exact reason for the missing correspondence. Possible reasons include payments by other payers (e.g., accident insurance) or imperfect cost type classification in the claims data.
