# Supplementary material for: Identifying diseases in claims data using a machine learning approach – a case from Switzerland
Source: Arch Public Health. 2025 Dec 30;83:320. doi: 10.1186/s13690-025-01813-y (PMC12750951; doi:10.1186/s13690-025-01813-y)
Supplement: Supplementary file 2 — Additional file 2. Details about hyperparameter tuning. [file 13690_2025_1813_MOESM2_ESM.pdf]

Additional file 2: Details about hyperparameter tuning

Article title: Identifying diseases in claims data using a machine learning approach – A case from Switzerland

Authors: Stucki, Kohler, Boes

```
#################
## Boosting
#################
library(gbm)

hyper_grid <- expand.grid(
  shrinkage = c(.01, .03, .05),
  interaction.depth = c(1,3,5),
  n.trees = (1:3)*1000,
  bag.fraction = c(.6, .8, 1),
)

tune_gbm_cv <- function(X, y, hyper_grid, cv_folds = 5) {

  # Create CV folds
  folds <- createFolds(y, k = cv_folds, list = TRUE)

  # Store results
  results <- data.frame()

  for (i in 1:nrow(hyper_grid)) {
    cat(paste("Testing combination", i, "of", nrow(hyper_grid), "\n"))

    # Extract parameters for this combination
    params <- hyper_grid[i, ]

    # Cross-validation scores for this parameter combination
    cv_scores <- matrix(numeric(cv_folds), nrow = 5, ncol = 2)

    for (fold in 1:cv_folds) {
      # Split data
      train_idx <- unlist(folds[-fold])
      val_idx <- folds[[fold]]

      X_train <- X[train_idx, ]
      y_train <- y[train_idx]
      X_val <- X[val_idx, ]
      y_val <- y[val_idx]
      traindata <- cbind(y_train, X_train)

      # Train model with current parameters
      set.seed(123)
      gbm_fit <- gbm(
        formula = y_train ~ .,
        data = traindata,
        distribution = "bernoulli",
        n.trees = params$n.trees,
```

```
    interaction.depth = params$interaction.depth,
    shrinkage = params$shrinkage,
    bag.fraction = params$bag.fraction,
    train.fraction = .75,
    verbose = FALSE
   )

   # Predict and compute metrics
   newdata <- cbind(y_val, X_val)
   predictions <- predict(gbm_fit, newdata = newdata,type="response",n.trees=params$n.trees)
   cv_scores[fold,1] <- PRAUC(y_pred=predictions,y_true=y_val)
  }

  # Store results for this parameter combination
  results <- rbind(results, data.frame(
   combination = i,
   shrinkage = params$shrinkage,
   interaction.depth = params$interaction.depth,
   n.trees = params$n.trees,
   bag.fraction = params$bag.fraction,
   mean_aucpr_cv = mean(cv_scores[fold,1]),
   stringsAsFactors = FALSE
  ))
 }
 return(results)
}
# Run hyperparameter tuning
cv_results <- tune_gbm_cv(mydata.train[, -1], mydata.train[, 1], hyper_grid, cv_folds = 5)

# Find best parameters
best_aucpr <- cv_results[which.max(cv_results$mean_aucpr_cv), ]
print("Best parameters:")
print(best_aucpr)

# View top 5 parameter combinations
print("Top 5 parameter combinations:")
print(head(cv_results[order(-cv_results$mean_aucpr_cv), ], 10))

position = which.max(cv_results$mean_aucpr_cv)
print(position)

# Fit final model using best parameters
set.seed(123)
boost.best <- gbm(
 formula = dep_var ~ .,
 data = mydata.train,
 distribution = "bernoulli",
 n.trees = cv_results$n.trees[position],
 interaction.depth = cv_results$interaction.depth[position],
 shrinkage = cv_results$shrinkage[position],
 bag.fraction = cv_results$bag.fraction[position],
 train.fraction = .75,
 verbose = FALSE
```

```
)

#################
## Random forest
#################

library(ranger)

number_Xvars <- dim(mydata.train)[2]-1
hyper_grid_rf <- expand.grid(
  mtry      = seq(2, number_Xvars, by = 6),
  node_size  = seq(1, 10, by = 9),
  samp_size = c(.33, .66, 1.0)
)

# total number of combinations
nrow(hyper_grid_rf)

# Hyperparameter tuning using cross-validation
tune_ranger_cv <- function(X, y, hyper_grid_rf, cv_folds = 5, num_trees = 500) {

  # Create CV folds
  folds <- createFolds(y, k = cv_folds, list = TRUE)

  # Store results
  results <- data.frame()

  for (i in 1:nrow(hyper_grid_rf)) {
    cat(paste("Testing combination", i, "of", nrow(hyper_grid_rf), "\n"))

    # Extract parameters for this combination
    params <- hyper_grid_rf[i, ]

    # Cross-validation scores for this parameter combination
    cv_scores <- matrix(numeric(cv_folds), nrow = 5, ncol = 2)

    for (fold in 1:cv_folds) {
      # Split data
      train_idx <- unlist(folds[-fold])
      val_idx <- folds[[fold]]

      X_train <- X[train_idx, ]
      y_train <- y[train_idx]
      X_val <- X[val_idx, ]
      y_val <- y[val_idx]

      # Train model with current parameters
      rf_model <- ranger(
        x = X_train,
        y = y_train,
        num.trees = num_trees,
        mtry = params$mtry,
        min.node.size = params$node_size,
```

```
      sample.fraction = params$samp_size,
      importance='impurity',
      probability=TRUE,
      seed= 123
    )

    # Predict and compute metrics
    newdata <- cbind(y_val, X_val)
    predictions <- predict(rf_model, data = newdata)
    cv_scores[fold,1] <- PRAUC(y_pred=predictions$predictions[,2],y_true=y_val)
  }

  # Store results for this parameter combination
  results <- rbind(results, data.frame(
    combination = i,
    mtry = params$mtry,
    min.node.size = params$node_size,
    sample.fraction = params$samp_size,
    mean_aucpr_cv = mean(cv_scores[fold,1]),
    stringsAsFactors = FALSE
  ))
 }

 return(results)
}

# Run hyperparameter tuning
cv_results <- tune_ranger_cv(mydata.train[, -1], mydata.train[, 1], hyper_grid_rf, cv_folds = 5,
num_trees = 500)

# Find best parameters
best_aucpr <- cv_results[which.max(cv_results$mean_aucpr_cv), ]
print("Best parameters:")
print(best_aucpr)

# View top 5 parameter combinations
print("Top 5 parameter combinations:")
print(head(cv_results[order(-cv_results$mean_aucpr_cv), ], 10))

position = which.max(cv_results$mean_aucpr_cv)
print(position)

rf_ranger <- ranger(
 formula       = dep_var ~.,
 data          = mydata.train,
 num.trees     = 500,
 mtry          = cv_results$mtry[position],
 sample.fraction = cv_results$sample.fraction[position],
 min.node.size   = cv_results$min.node.size[position],
 importance='impurity',
 probability=TRUE
)
```
